# Synopsis of Discussions, Missouri State Dental Society

**Published:** 1866-05

**Authors:** A. M. Leslie


### SYNOPSIS OF DISCUSSIONS OF MISSOURI STATE DENTAL SOCIETY.

Reported by request of the Society, by A. M. Leslie, D. D. S.

(CONTINUED FROM PAGE 185.)

DR. CLARK—Gentlemen seem to misconceive his ideas. He is convinced we frequently call that absorption which is not absorption. There are two very distinct causes of absorption. One is pressure. The effects of a grain of sand placed under the gum will illustrate this class. Most foreign substances lodged in the living tissues, will, in general, result in necrosis, more or less extensive. Does not suppose this result always is reached through inflammation.

The only good treatment in such cases is, first, to remove all foreign substances. Failing in that you fail in the case. Do that, and ninety-nine hundreths will recover.

In the other cause of this disease, (absorption of the alveolus,) he was still more in the dark. Finds, here, the margin of the gum and the periosteum inflamed. In some such cases, has never succeeded in subduing the inflammation. What the irritant is that produces this inflammation, he knows not.

DR. HOVEY—Has no doubt absorption of the alveolus is the result of some infringing substance. Tartar not only produces inflammation and absorption of gum, but goes on until it infringes on the alveolar processes. It follows up so as to reach the points of the roots, in some cases. He thinks that in the cases referred to by Dr. Judd, an acid will be found to be present, which is the result of pus. He had a set of teeth made for his own use. During their use, as absorption progressed, foreign substances would accumulate on them, sufficient in three days to acidify a pint of water.

DR. JUDD—In some cases absorption goes on when there is almost no disease in the gums. When they are turgid, could account for it as a case of scurvy. But had seen absorption existing where there was no tartar, and the saliva was bland. Syphilitic origin may be suspected when the whole mucous membrane is turgid.

Dr. Clark—For arresting the disease, trusts to astringents and cleansing. Is at a loss in treating cases when these are not indicated. Has tested with litmus paper, and generally found the saliva free of acid. Probably not one in ten showed an acid re-action. Has tried alkalies, astringents, creosote, and nit-argenti. The last being most successful,—but its use is apt to cause absorption of the gum. It causes union at the point where tartar ceases, but the free portion is destroyed. Finds in these cases an irritated state of the neck of the tooth. Objects to all applications to it.

Dr. Payne—Had a first bicuspid in the second condition and could not touch the neck with a brush. Applied creosote and arsenic and restored the gum. Tooth now good.

Dr. McKellops—Has failed in the second class of cases with all kinds of treatment. One case was a lady, aged 25. Had perfect teeth which she took great care of. He could see nothing the matter with the gum. The gum over the right central incisor began to recede. There was no soreness, no elongation. At times there was a little discharge. Tried, effectually, the big and little nigger of Dr. Atkinson's Pharmacy, but found injury only to result. Cannot account for this case. Dr. Morse reports to him that he has seen the case, and the absorption progresses and that the tooth will be lost.

Another case is a gentleman, aged 50. Takes great care of his teeth, which have a transparent appearance. Consults his dentist regularly. Finds no deposit. On pressure of the gum there is, at times, a slight effusion. At times, the mouth is very healthy. General absorption, however, is going on around the six front teeth. There is no tenderness of the necks of the teeth. Has no doubt the disease has a syphilitic origin. Another case was one in which the party went to Europe to be treated. There he was treated rigidly as for syphilis. Portions of the superior maxille were so dressed that a probe could be passed through them. Had several teeth extracted and was ultimately cured, with the loss of but a small portion of the hard palate. He underwent the treatment in a German hospital, especially devoted to the treatment of syphilitic diseases.

Dr. Hale, Jr.—Had a similar case to the second mentioned by Dr. McK. Dr. McClurg, of Ky., recommended the use of crocus and alcohol. This seemed to act like a charm, as the case got well. Has used it since with good effect.

Dr. Wilson—When practicing in Rochester, in 1848, a lady complained of pain in second lower molar. Found no cause for tooth-ache, and declined to extract. In two months she again insisted on extraction. Still declined. Thought he now discovered a slight opacity. He declined a third time to extract, but she returned afterwards with the tooth in her hand. He probed the process and found it quite porous. He since extracted two teeth for her.

Dr. Clark—Has seen a case, something like this, result from trying to break a hickory nut. The result was necrosis.

Dr. Judd—Has seen a few cases like these. Supposes the pathological conditions the same. Extracted, in one case, seven teeth followed by an operation. Has seen cases where a small loss occurred. In other cases, osteo sarcomo has resulted. Some of the most difficult surgical diseases have their location in the mouth and face. One case with the gum much inflamed, had been treated several months by physicians. On examination, he concluded it was scurvy, and treated it with lemonade, producing a cure in three weeks. Had seen one child, 4 years old, with ulceration around four superior incisors. Treated this case locally with nitrate of silver, and constitutionally with stimulants and tonics.

Found, in some cases, that the use of nit-argenti before the use of stimulants and tonics was injurious. In such cases he procured the effect of the stimulants and tonics first, after this found the N. Scl. efficacious.

Dr. Hovey—Thinks these difficult cases must be scrofulous, and approved of the constitutional treatment.

Dr. Prevost—Gave a case of a child in Ohio, 7 years old. Suffered long with left side of lower jaw. Probed and found a necrosed piece of bone, loose. First and second molar both diseased, extracted them and the necrosed portion, and the recovery was good.

Dr. Eames—Has had two cases in which he used the "big nigger." One was a gentleman with four incisor teeth exposed, in part. One quite loose, and could be removed easy.

Under this treatment is now doing well. Another was a lady with loose teeth, which are now doing well. In other cases, has found variations. Some improve and some fall back again to the former condition. Two or three applications generally restore to health, in favorable cases.

In the first case he cut down to the point of the fang of the very loose tooth, that he might clean it thoroughly.

Dr. McKellops—While in New York, looked in on Atkinson. Saw several cases he was treating. They were all syphilitic. Some were affected in the mouth, others in the face, others had sores on the legs. He treats them constitutionally, and gives them peculiar baths. He tried a bath on himself. After a thorough opening of the pores of the skin, by the application of warm vapors, he rubs with a Russian towel. He then thoroughly washes the skin with lemon juice, which is followed by annointing with pure olive oil. To Atkinson he sent a leading opera singer, who had a dead molar tooth, with one fang denuded. This was pronounced a syphilitic case.

Atkinson was supposed to have been very successful in the treatment. He afterwards met this patient in Paris, and found the tooth still troubling him. He found the fang still denuded. He finds the preparations of iodine and creosote useful as local applications for soreness.

Dr. Judd—Desired to know what is the principle involved in the use of Atkinson's solutions. Supposes they act on the same principle as made use of in the closure of cleft palate, by action in the edges of the surrounding membrane.

ALVEOLAR ABSCESS.

DR. FORBES—In alveolar abscess he sends the patients home to apply water, as hot as bearable, and directs brushing the gums until they cease to bleed. Then removes the tartar. In young patients this is generally enough. If pus is present, he uses the iodine preparations and constitutional treatment. If the patient is past the meridian of life, considers success uncertain. The hope then of restoring the process, if absorbed, is very small. Has never succeeded in accomplishing it. Does not believe the *bone* has been restored. He wants to see this. If the process can be raised one line, it can be raised two. If two, why then it can be to the edge of the gum. Remove the cause, and nature with a little stimulant, restores the loss, unless membranes are removed.

DR. HALE, JR.—In answer to Dr. Forbes' question, "Is the fashionable mode of drilling into a tooth to relieve abscess" yet followed, would say, his father for thirty-five years has practiced it. He thinks it practicable, if not fashionable. Thinks it good practice when a tooth gives great pain two weeks after filling.

DR. MORSE—Thinks the perforating to relieve pain is not good practice. If the nerve is dead should be removed and fang filled. Had a tooth in his mouth drilled and found it very offensive until removed.

DR. HALE, JR.—Can show many cases in his father's practice where there exists no continuous discharge.

DR. MCKELLOPS—Considers it no better to drill a hole and leave it open, than to remove a plug and leave the cavity open. He removes the filling and treats the fang. A case he saw in an office in Paris, was a central incisor in gent's mouth. A knitting needle could be passed up between the teeth. Tried Atkinson's preparation first; then recommended phenic acid, the first application of which removed all smell. The opening in the gum finally closed and the tooth was preserved. Considered it a very difficult case to treat. Has great success in alveolar abscess over the front teeth. Admits the hole made in the tooth will relieve from pressure, but the smell remains. In plugging fang prefers gold to the point.

Dr. Clark—A surgeon sets out to extirpate, as far as he can, obstructions to nature. The dental surgeon should not stop short of the same. Like Dr. McKellops, his hobby is to treat dead teeth. In early days has filled teeth which ulcerated. Afterwards has drilled to give relief. This was a great disgust to him, as, although he gave relief, he considered that tooth a dead one. When he went to New Orleans, he rejoiced to get off from those pest houses. In New Orleans he resolved he would take time in every case. Will not refer, here, to the origin of removing the pulp, (that he has done elsewhere), and leaving a clean thing in the mouth. Looks back with pleasure to that part of his practice. No dentist has a right to stop short of doing all he can to save any tooth. Is glad the dentists of Missouri are doing all they can to remove all diseases in their field. In cases where there are sacks on the point of fangs, he generally treats through the alveoli. First, however, he tries what can be done through the fang. He has heard much about restoration of processes, but has yet to see the first case of such restoration. In cases he has spoken of, there does seem to be a filling up. But he believes if we cut down we will find all such cases are not filled with bone.

Dr. Rivers—Was consulted by a young lady. Found her right superior literal incisor loose, gum and lip swollen. Found no decay. Other teeth good. Would not allow drilling, as pressure hurt. He would not extract—it suppurated. In four days was able to drill into it. He then applied all Atkinson's preparations. Has treated it six months, and, as yet, no success.

Dr. Clark—Referred to difficulty in lateral incisors in such cases.

Dr. Forbes—Considers the lower molars the most difficult cases to treat—attributes this to the gravity of the pus in the fang. He treats these by syringing out clean, then wipes out as dry as possible; uses, cotton and creosote, (the first as a non-conductor), pressing as near point of fang as possible. Thinks his old plan of using cotton moistened with creosote and then dried, as a non-conductor, possibly better. He invariably fills the fang above the cotton with gold.

Dr. Clark—Cannot see any bad effect produced in the surrounding parts by filling the entire fang with metal. No objections to use of creosote in preparing such teeth, the only use he has for a non-conductor is in living teeth. In some cases this change of temperature may be of some use, as a douche is of use to the body. Thinks it benefits some teeth which are filled. Has ceased experimenting with new materials for filling, since he was satisfied gold well put in a cavity will preserve a tooth at that point. Believes the nerve may be extracted from 1-16 to 1-8 inch beyond point of fang.

Dr. Forbes—Believes we do not remove beyond point of fang.

Dr. Hovey—Believes the fangs still possessed of some life. Therefore, it may be proper to fill them with a non-conducting material. Uses cotton and creosote at the point of fang, and thus tans the part. Enlarges the crown cavity, but thinks it best not to enlarge the fang cavity. Finds great difficulty in buccal fangs of superior molars, and the fangs of lower molars. Syringes thoroughly. Thinks about twenty-five per cent. of these cases have to be re-treated.

Dr. Clark—Is well aware the fangs are held in by living tissues, and does not consider a fang a foreign body; still he considers it not sensitive to the change of temperature. Is certain he can remove a portion of pulp greater than the length of the canal.

Dr. Forbes—The advancement of science is the expression of what has been discovered. Has seen a man three days on the witness stand learning to tell *only* what he does know.

Dr. Blake—Uses cotton at the point of fang, because it more perfectly closes the foramen.

Dr. Morrison—Thinks cotton can be carried to the point of the fang with a fine broach. Considers the theory of a non-conductor in the fang as good. Uses it and creosote, but not as a non-conductor. Thinks them a good filling for fangs of lower teeth. Uses creosote to make an insoluble compound with the albumen.

Dr. McCoy—Asks older practitioners how they reach front fangs of second sup. molar, with the cavity in the posterior approximal surface.

Dr. Forbes—Does not get into them, does not cut away, does not drill; trusts to the effect of creosote.

Dr. Eames—Would drill. Cuts away the cavity, and gets into the cavity if possible.

Dr. McCuen—Is satisfied with removing the pulp in main cavity, and gets into the fangs as far as possible.

Dr. McKellops—Does the best he can in such cases. He cuts perfectly through the crown. Cuts right down in the cavity, enlarging it until he reaches the fangs, and trusts to restoring the form by building up with gold. If he cannot get into the fangs he extracts the tooth. In the case of the singer he spoke of sending to Atkinson, he found A. had cut through the sound portion of crown to get at the fangs, to clean them. He (McK.) does not fill until all irritation is removed. Sometimes this takes sixteen weeks.

